# Genetic Polymorphisms in Inflammasome-Dependent Innate Immunity among Pediatric Patients with Severe Renal Parenchymal Infections

**DOI:** 10.1371/journal.pone.0140128

**Published:** 2015-10-07

**Authors:** Chi-Hui Cheng, Yun-Shien Lee, Chee-Jen Chang, Jui-Che Lin, Tzou-Yien Lin

**Affiliations:** 1 Division of Pediatric Nephrology, Department of Pediatrics, Chang Gung Children’s Hospital, Chang Gung Memorial Hospital, Taoyuan, Taiwan; 2 College of Medicine, Chang Gung University, Taoyuan, Taiwan; 3 Genomic Medicine Research Core Laboratory, Chang Gung Memorial Hospital, Taoyuan, Taiwan; 4 Department of Biotechnology, Ming-Chuan University, Taoyuan, Taiwan; 5 Statistical Center for Clinical Research, Chang Gung Memorial Hospital, Taoyuan, Taiwan; 6 Institute of Oral Medicine, Department of Chemical Engineering, National Cheng Kung University, Tainan, Taiwan; 7 Division of Pediatric Infectious Diseases, Department of Pediatrics, Chang Gung Children’s Hospital, Chang Gung Memorial Hospital, Taoyuan, Taiwan; University of Utah School of Medicine, UNITED STATES

## Abstract

**Background:**

Inflammasome innate immune response activation has been demonstrated in various inflammatory diseases and microbial infections. However, to our knowledge, no study has examined the inflammasome-dependent pathways in patients with urinary tract infection. Defective or variant genes associated with innate immunity are believed to alter the host’s susceptibility to microbial infection. This study investigated genetic polymorphisms in genes encoding inflammasomes and the subsequent released cytokines in pediatric patients with severe renal parenchymal infections.

**Methodology:**

This study included patients diagnosed with acute pyelonephritis (APN) and acute lobar nephronia (ALN) who had no underlying disease or structural anomalies other than vesicoureteral reflux (VUR). Single nucleotide polymorphism (SNP) genotyping was performed in the genes associated with inflammasome formation and activation (*NLRP3*, *CARD8*) and subsequent IL–1β cytokine generation (*IL–1β*).

**Principal Findings:**

A total of 40 SNPs were selected for initial genotyping. Analysis of samples from 48 patients each and 96 controls revealed that only nine SNPs (five SNPs in *NLRP3*; three SNPs in *CARD8*; one SNP in *IL–1β*) had heterozygosity rates >0.01. Hardy–Weinberg equilibrium was satisfied for the observed genotype frequencies of these SNPs. Analysis excluding patients with VUR, a well-known risk factor for severe UTIs, revealed a lower frequency of the CC genotype in *NLRP3* (rs4612666) in patients with APN and ALN than in controls. Correction for multiple-SNP testing showed that the non-VUR subgroup of the APN+ALN combined patient groups remained significantly different from the control group (*P* < 0.0055).

**Conclusions:**

This study is the first to suggest that the inflammasome-dependent innate immunity pathway is associated with the pathogenesis of pediatric severe renal parenchymal infections. Further investigation is warranted to clarify its pathogenic mechanism.

## Introduction

Urinary tract infections (UTIs) are among the most prevalent bacterial infectious diseases among pediatric patients, with morbidity risks of ~3% and ~1% in prepubertal females and males, respectively [1]. The clinical severity of UTIs ranges from uncomplicated lower UTIs, such as asymptomatic bacteriuria (ABU), to acute pyelonephritis (APN), acute lobar nephronia (ALN), and renal abscess formation [2, 3]. Among these UTIs, ALN presents as a localized non-liquefactive inflammatory renal bacterial infection. ALN has been identified as a complicated form of acute renal infection, representing progression of the inflammatory process of APN. Our previous studies suggested that ALN might represent a relatively early stage in renal abscess development [2, 3], with a longer duration of antibiotic treatment recommended for patients with ALN compared with those with APN [2]. Nevertheless, APN, ALN, and intrarenal abscess are considered the most serious forms of UTIs requiring different durations of antibiotic treatment. Furthermore, in some cases, additional surgical procedures are indicated for proper treatment [2, 4].

Patients’ susceptibility to UTIs and disease severity are determined by complicated interactions between the host and pathogenic microbes [4–6]. In addition to certain virulence factors associated with uropathogenic *Escherichia coli* in specific UTIs [4–8], intra-individual variations play a role in clinical severity. These observations highlight the importance of host factors, such as the presence of vesicoureteral reflux (VUR) or genetic polymorphisms in innate immunity pathway genes, in defending bacterial invasion and infection [9–11].

The innate immune system is considered the first line of defense against microbial infection. In response to microbial invasion, innate immunity is initiated or activated with expression of extracellular or intracellular germline-encoded pattern-recognition receptors (PRRs) [12, 13]. Next, various intracellular signaling cascades are triggered, which ultimately culminate in host defense responses to eliminate the microbial invasions. However, such host responses can also result in various tissue injuries if the host proinflammatory responses are too robust.

Previously, we studied pediatric patients with UTIs of differing clinical severity to examine the associations with genetic variations in the innate immunity pathways that were dependent upon Toll-like receptors (TLRs), one of the best-characterized PRRs. We demonstrated that the AA genotype and A allele of the *IL–8* SNP were related to patient susceptibility to parenchymal infection and were correlated with the severity of infection in pediatric patients with APN and ALN, probably due to the upregulation of IL–8 expression [14]. Additionally, MALDI-TOF SNP analyses revealed that the genetic variant in *TLR–2* (rs3804100, T1350C) might protect the host from severe UTIs such as ALN [15].

Similar to TLRs, the cytosolic nucleotide-binding domain–leucine-rich repeat-containing receptors (NLRs, or NOD-like receptors) have been investigated as PRRs for microbial attacks [13, 16, 17]. In addition to recognizing the pathogen-associated molecular patterns (PAMPs), the highly conserved markers specific to microbes, these PRRs can also sense danger signals or danger-associated molecular patterns (DAMPs) released after cellular damage or stress induced by pathogen attacks [13, 16, 18]. Pathogen recognition activates the host inflammasome, a complex of cytosolic NLR-containing proteins, including the NLRP3 protein, protease caspase–1, and the adaptor protein ASC (apoptosis associated speck-like protein containing a CARD; the caspase recruitment domain). Activated caspase–1 leads to the cleavage of the pro-IL–1β and pro-IL–18 to produce IL–1β and IL–18 cytokines, respectively, to form an inflammasome-dependent immune response [17, 19, 20].

Despite of inflammasome-dependent immune response has been reported to be associated with various microbial sensing and reaction to microbial infections [13, 16, 21, 22], none has explored its likely association with urinary tract infections, to our knowledge. Henceforth, to exam inflammasome-dependent innate immunity pathway on pediatric patients with severe renal parenchymal infections might provide supplementary information to our earlier findings based on the TLR-dependent pathway [14, 15]. In this investigation, we attempted to assess the likely variations in genes associated with the inflammasome-dependent innate immunity pathway for pediatric patients with various severe UTIs. The genes selected for initial genotyping involved in inflammasome formation (*NLRP3*, *CARD8*) and subsequent activation and IL–1β cytokine generation (*IL–1β*). Among these, *NLRP3* has been implicated to be associated with the tubulointerstitious disease, such as renal infection [19]. These components may play a role in various inflammatory responses and bacterial clearance in pediatric patients with UTIs of differing clinical severity, such as APN and the clinically more severe disease ALN. Additionally, as VUR is a well-known risk factor for severe parenchymal infectious disease [9, 23], a subgroup of APN and ALN cases without VUR (non-VUR) was examined to exclude the possible effects of VUR.

This investigation was part of our ongoing effort to explore the pathogenic host and bacterial urovirulence factors related to pediatric APN and ALN; as such, it includes patients continuously enrolled since our previous studies [5, 14, 15]. Therefore, compared with our previous reports, this study enrolled a greater number of participants, with a total of 138 APN and 222 ALN cases and 225 controls, including those cases in our earlier investigations on SNPs in TLR-dependent innate immunity pathway genes (113 APN and 172 ALN cases and 222 controls) [14, 15]. Genotying for the genes associated with the inflammsome-dependent pathway was commenced on the second half of year 2012, while genotyping for genes associated with the TLR-dependent pathway presented in earlier publications [14, 15] has been completed in 2010.

## Materials and Methods

### Ethics statement

This investigation was approved by the Institutional Review Board of Chang Gung Memorial Hospital. Written informed consent was obtained from the parents of all participating patients following a full explanation of the study.

### Study setting and patient selection criteria

This study is part of our ongoing investigation of the pathogenic host and bacterial urovirulence factors related to pediatric APN and ALN [5, 14, 15]. Patients were enrolled in the study if they fulfilled the diagnostic criteria for APN and ALN caused by *E*. *coli* and lacked any of the exclusion criteria. Participants were admitted to Chang Gung Children’s Hospital, a tertiary medical center located near Taipei in northern Taiwan, between January 2004 and December 2008 as well as between January 2012 and December 2013. The controls consisted of patients who visited the outpatient clinic for reasons other than a UTI or severe infection. The controls had no history of UTI or severe infections and no positive urine culture [14, 15].

A detailed diagnostic schematic plan for patients suspected of having APN or ALN was described previously [2, 5, 14, 15]. Briefly, all patients with a suspected UTI due to the presence of pyuria (>5 white blood cells/high-power field) who had fever with symptoms and signs related to UTIs (*e*.*g*., pain, dysuria, and frequent urination) or without focus underwent renal ultrasonography on the first or second day after admission. Computed tomography (CT) was performed immediately when the initial ultrasonographic findings showed unilateral or bilateral nephromegaly or a focal renal mass. For patients who presented with borderline nephromegaly on ultrasonography, CT was performed when the patient remained febrile for 72 h after starting the antibiotic therapy. ALN was diagnosed based on positive CT findings. Technetium 99m-dimercaptosuccinic acid scintigraphy (^99m^Tc-DMSA) was performed within 3–7 days of admission in patients suspected of having a febrile UTI who did not satisfy the sonographic criteria for ALN. APN was defined as focal or diffuse areas of decreased ^99m^Tc-DMSA uptake without evidence of cortical loss.

Patients were excluded from this study if they showed evidence of underlying disease such as diabetes; immunodeficiency; or structural anomalies such as neurogenic bladder, posterior urethral valve, urinary diversion, bladder diverticulum, ureterocele, or urinary tract obstruction (other than VUR).

### Genotyping by matrix-assisted laser desorption/ionization time-of-flight (MALDI-TOF)-based mini-sequencing analysis or direct DNA sequencing

Genotyping for the genes associated with inflammasome-dependent innate immunity pathway presented here commenced on the second half of year 2012, while genotyping for genes associated with the TLR-dependent pathway presented in earlier publications [14, 15] has been completed in 2010. No further genotyping on the inflammasome-dependent pathway was planned.

SNPs in the genes encoding NLRP3, CARD8, and IL–1β, as well as in their respective promoter regions, were identified in the NCBI dbSNP database [24]. A total of 40 SNPs (rs35829419, rs10754558, rs4925659, rs12239046, rs10754555, rs35829479, rs10733113, rs1539019, rs4925648, rs4925663, rs10802501, rs2027432, rs76291085, rs4353135, rs4266924, rs55646866, rs6672995, rs4925650, rs3806265, rs4612666, rs10733112, rs12079994, rs12048215, rs10925019, rs4925654, rs10925026, rs12565738, and rs4378247 for *NLRP3*; rs2043211, rs6509365, rs1965759, rs1062808, rs4389238, rs2288877, rs2288876, and rs1972619 for *CARD8*; rs1143643, rs1143634, rs1143629, and rs16944, for *IL–1β*) were selected for initial genotyping based on previous studies on SNPs in inflammasome-dependent pathogenic mechanisms for various diseases [25–42]. Analysis of samples from 96 controls and 48 patients each revealed that only nine SNPs had heterozygosity rates >0.01. Among these, rs4612666 (*NLRP3*), rs4925650 (*NLRP3*), rs10754558 (*NLRP3*), rs1965759 (*CARD8*), rs2043211 (*CARD8*), and rs1143629 (*IL–1β*) were analyzed by direct DNA sequencing (S1 Table). Three other SNPs, rs1539019 (*NLRP3*), rs4925663 (*NLRP3*) and rs1972619 (*CARD8*), were genotyped by matrix-assisted laser desorption/ionization time-of-flight (MALDI-TOF)-based mini-sequencing analysis (S2 Table).

Genomic DNA was extracted from peripheral blood lymphocytes using a Nucleospin^®^ Blood DNA extraction kit (Macherey-Nagel, Düren, Germany) [15]. For direct DNA sequencing, polymerase chain reaction (PCR) was performed in a master mix (50 μL) containing 25 ng of DNA, 25 μL DreamTaq Green PCR Master Mix (2x) (Thermo Scientific), 0.5 μL of each primer (5 pM, Integrated DNA Technologies, IDT) (S1 Table), and 23.5 μL distilled deionized water (dd H_2_O). PCR was carried out for 35 cycles with initial denaturation at 94°C for 2 min followed by denaturation at 94°C for 30 s, annealing for 30 s, extension at 72°C for 40 s, and final extension at 72°C for 7 min. All PCR products were analyzed on a 1.5% agarose gel to verify the amplification reaction. The amplified products were then subjected to direct automated sequencing (ABI Prism 3730 DNA Analyzer).

For MALDI-TOF-based mini-sequencing analysis, the SNPs were genotyped as described previously [14, 15] using the primers listed in S2 Table. Briefly, the PCR mix (25 μL) contained 200 ng of genomic DNA, primers (25 pM each), dNTPs (0.2 mM), 1× Fast-Start PCR buffer, 1 M betaine, and 1 U of Fast-Start Taq Polymerase (Roche Diagnostics, Basel, Switzerland). The PCR reaction was initiated at 95°C for 5 min, followed by 40 cycles at 95°C for 45 s, 50°C for 45 s, and 60°C for 45 s, with a final extension at 52°C for 10 min. Unincorporated dNTPs and primers were removed automatically by MAPIIA (GenePure PCR Purification System; Bruker, Bremen, Germany). The purified products were amplified further using the respective mini-sequencing primers (S2 Table) in 20 μL of a solution containing 50 ng of the PCR product, 1 μL (10 pmol) of mini-sequencing primer, 0.5 μL of 1 mM ddNTP/dNTP mixture, 0.5 U of Thermo Sequenase DNA Polymerase (Amersham Biosciences, Piscataway, NJ), and 2 μL of the reaction buffer provided by the manufacturer. The reactions were carried out with initial denaturation at 96°C for 1 min, followed by 50 cycles of 96°C for 15 s, 50°C for 15 s, 60°C for 100 s, and 96°C for 30 s (Thermo Hybaid, Waltham, MA). The reaction products were purified automatically by MAPIIA (Single-Strand DNA Binding Beads; Bruker). The purified samples were then mixed with 0.5 μL of matrix solution (50 mg/mL 3-hydropicolinic acid in a 4:5:1 mixture of water, acetonitrile, and 50 mg/mL diammonium citrate) and spotted onto 384-well Teflon sample plates (PerSeptive Biosystems, Framingham, MA). MALDI-TOF mass spectra were acquired with a Bruker Autoflex MALDI-TOF mass spectrometer (Bruker) and AutoXecute software (Bruker) to validate the genotype data.

### Statistical analysis

Hardy–Weinberg equilibrium (HWE) was tested for goodness-of-fit using a χ^2^ test with one degree of freedom. Comparison of genotype and allele frequencies among the control, APN, and ALN groups were performed by χ^2^ analysis or two-sided Fisher’s exact test, as appropriate. The association of outcome and SNP genotype was analyzed with recessive and dominant models [10]. For the dominant model, the genotypes of minor homozygotes and heterozygotes were combined and compared with common homozygous genotypes. In a similar manner, the genotypes of common homozygotes and heterozygotes were combined and compared with minor homozygous genotype for recessive model analysis. A *P*-value <0.05 was considered to indicate statistical significance. As no adjustments were made to correct for multiple comparisons, the chance of type I errors may have been increased. All statistical analyses were performed using SPSS software (Version 17.0, IBM SPSS Statistics).

At first, 96 cases (48 APN and 48 ALN) and 96 control samples were analyzed for *NLRP3* (rs4612666) SNP. These 192 samples indicated that the probability of exposure among controls is 0.25. Using the dominant model, if the true odds ratio for disease in exposed subjects relative to unexposed subjects is 1.89 as we noted among these 192 samples, we will need to study 196 patients and 196 control patients to be able to reject the null hypothesis that this odds ratio equals 1 with probability (power) of 0.8. The Type I error probability associated with this test of this null hypothesis is 0.05. The samples size estimate was performed with PS Power and Sample Size Calculations Version 3.1.2 (http://biostat.mc.vanderbilt.edu/PowerSampleSize) [43].

## Results

A total of 360 patients (182 males and 178 females) who fulfilled the enrollment criteria for APN and ALN were included in this study. Among these, 138 patients (77 males and 61 females; 2.50 ± 2.98 years old; range, one month to 14.42 years old) were diagnosed with APN and 222 (105 males and 117 females; 3.05 ± 2.78 years old; range, one month to 15 years old) with ALN. The control population consisted of 225 subjects (123 males and 102 females; mean age ± SD, 2.96 ± 3.02 years old; range, one month to 11 years old]). Although more patients were included in this investigation as compared to those reported in earlier studies [14, 15], statistical differences in the demographic and clinical characteristics among these three groups remained similar to those reported earlier [14, 15], with no significant differences detected in the sex ratio and age among the three groups (control, APN and ALN).

**Fig 1:**
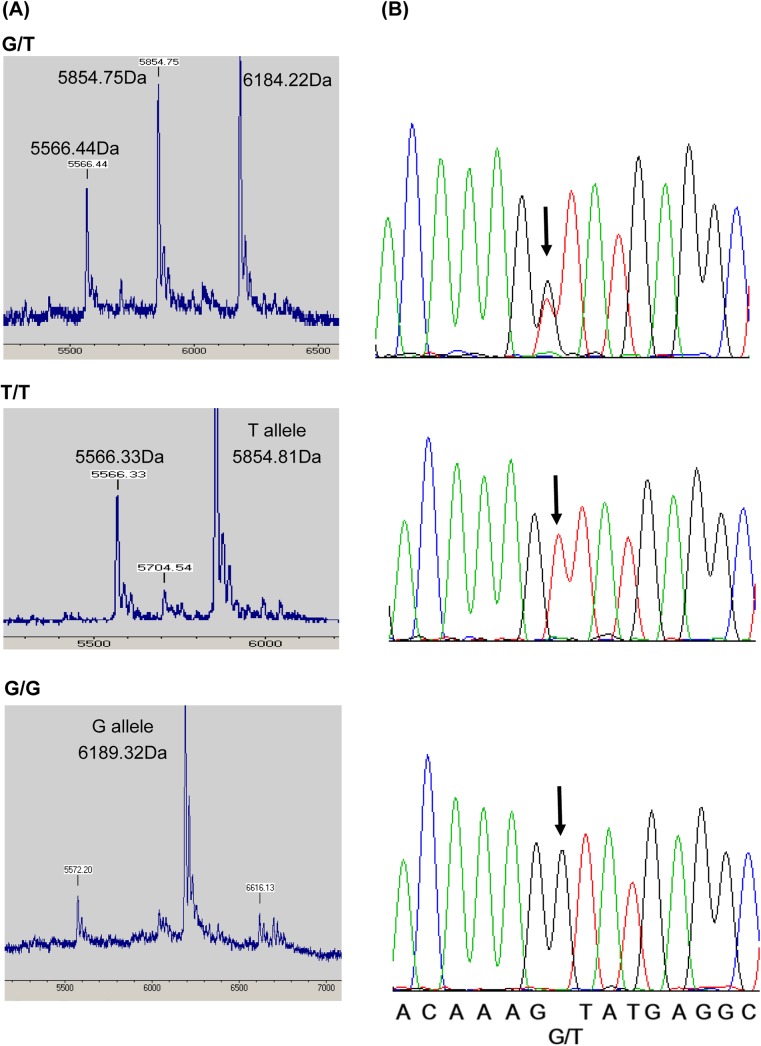
MALDI-TOF mass spectra from the genotyping of *NLRP3* (rs1539019) PCR product and its sequencing results. (A) The SNPs were genotyped by MALDI-TOF MS based on the molecular weights of the mini-sequencing products listed in S2 Table. (B) Sequencing results for each of the PCR products from the G/T, T/T, and G/G genotypes of rs1539019. The SNPs are indicated by arrows.

The observed genotype frequency in all groups met the requirements for HWE. The MALDI-TOF mass spectra in *NLRP3* (rs1539019) matched with the auto-sequencing results from randomly selected cases (Fig 1), indicating that MALDI-TOF could be used as the direct DNA sequencing method for analyzing the likely SNPs.

Genotypic analyses of the nine SNPs revealed that *NLRP3* (rs4612666) demonstrated a significant difference in genotype frequency between the APN and control cases [OR (95% CI): 2.32 (1.66, 3.97)] as well as between the APN and ALN cases [OR (95% CI): 0.50 (0.29, 0.86)] in the dominant model (Table 1). Additionally, *NLRP3* (rs4925650), *NLRP3* (rs10754558), and *CARD8* (rs1965759) showed significant differences among different groups using the recessive model (Table 1). Nevertheless, after correction for multiple-SNP testing (nine SNPs examined), only *NLRP3* (rs4612666) showed a significant difference between the APN and control using the dominant model (*P* < 0.0055). Allele frequency analyses demonstrated that *NLRP3* (rs4612666) and *NLRP3* (rs10754558) showed significant differences when comparing ALN with APN [OR (95% CI): 1.42 (1.04, 1.93)] and APN with control [OR (95% CI): 1.46 (1.20, 2.01)] (Table 2).

**Table 1:** Genotypic analysis of the SNPs.

SNP	Group	Genotype, *n* (%)			Dominant model				Recessive model			
		00	01	11	(01, 11 *vs*. 00)				(00, 01*vs*. 11)			
		CC	CT	TT	OR (95% CI)	*P* ^a^	OR (95% CI)	*P* ^a^	OR (95% CI)	*P* ^a^	OR (95% CI)	*P* ^a^
*NLRP3*	Control	68 (32.7)	95 (45.7)	45 (21.6)	1.00				1.00			
(rs4612666)	APN	23 (17.3)	84 (63.2)	26 (19.5)	**2.32 (1.66, 3.97)**	**0.002** ^c^	1.00		1.14 (0.66, 1.95)	0.644	1.00	
	ALN	61 (29.5)	116 (56.0)	30 (14.5)	1.16 (0.77, 1,76)	0.478	**0.50 (0.29, 0.86)**	**0.011**	1.63 (0.98, 2.71)	0.059	1.43 (0.81, 2.55)	0.220
	Combined^b^	84 (24.7)	200 (58.8)	56 (16.5)	1.48 (1.01, 2.17)	0.043			1.40 (0.90, 2.17)	0.133		
		GG	GA	AA	OR (95% CI)	*P*	OR (95% CI)	*P*	OR (95% CI)	*P*	OR (95% CI)	*P*
*NLRP3*	Control	66 (31.9)	97 (46.9)	44 (21.3)	1.00				1.00			
(rs4925650)	APN	40 (30.5)	70 (53.4)	21 (16.0)	1.07 (0.66, 1.71)	0.794	1.00		1.41 (0.80, 2.51)	0.235	1.00	
	ALN	65 (30.4)	89 (41.6)	60 (28.0)	1.07 (0.71, 1.62)	0.738	1.01 (0.63, 1.62)	0.975	0.69 (0.44, 1.08)	0.107	**0.49 (0.28, 0.85)**	**0.011**
	Combined	105 (30.4)	159 (46.1)	81 (23.5)	1.07 (0.74, 1.55)	0.721			0.88 (0.58, 1.33)	0.546		
		CC	CG	GG	OR (95% CI)	*P*	OR (95% CI)	*P*	OR (95% CI)	*P*	OR (95% CI)	*P*
*NLRP3*	Control	85 (37.9)	105 (46.9)	34 (15.2)	1.00				1.00			
(rs10754558)	APN	64 (47.8)	59 (44.0)	11 (8.2)	0.67 (0.43, 1.03)	0.066	1.00		**2.03 (0.99, 4.16)**	**0.049**	1.00	
	ALN	86 (40.0)	102 (47.4)	27 (12.6)	0.92 (0.63, 1.35)	0.659	1.37 (0.89, 2.12)	0.151	1.25 (0.72, 2.15)	0.427	0.61 (0.29, 1.28)	0.189
	Combined	150 (43.0)	161 (46.1)	38 (10.9)	0.81 (0.58, 1.14)	0.228			1.47 (0.90, 2.42)	0.124		
		GG	GT	TT	OR (95% CI)	*P*	OR (95% CI)	*P*	OR (95% CI)	*P*	OR (95% CI)	*P*
*NLRP3*	Control	86 (39.4)	104 (47.7)	28 (12.8)	1.00				1.00			
(rs1539019)	APN	53 (40.5)	58 (44.3)	20 (15.3)	0.96 (0.62, 1.49)	0.852	1.00		0.82 (0.44, 1.52)	0.524	1.00	
	ALN	67 (32.2)	104 (50.0)	37 (17.8)	1.37 (0.92, 2.04)	0.120	1.43 (0.91, 2.25)	0.122	0.68 (0.40, 1.16)	0.156	0.83 (0.46, 1.51)	0.546
	Combined	120 (35.4)	162 (47.8)	57 (16.8)	1.19 (0.84, 1.69)	0.334			0.73 (0.45, 1.19)	0.203		
		TT	TC	CC	OR (95% CI)	*P*	OR (95% CI)	*P*	OR (95% CI)	*P*	OR (95% CI)	*P*
*NLRP3*	Control	57 (28.9)	97 (49.2)	43 (21.8)	1.00				1.00			
(rs4925663)	APN	29 (24.8)	60 (51.3)	28 (23.9)	1.24 (0.73, 2.08)	0.426	1.00		0.89 (0.52, 1.53)	0.666	1.00	
	ALN	51 (24.8)	101 (49.0)	54 (26.2)	1.24 (0.80, 1.92)	0.344	1.00 (0.59, 1.69)	0.995	0.79 (0.50, 1.24)	0.303	0.89 (0.52, 1.50)	0.651
	Combined	80 (24.8)	161 (49.8)	82 (25.4)	1.24 (0.83, 1.84)	0.295			0.82 (0.54, 1.25)	0.357		
		CC	CT	TT	OR (95% CI)	*P*	OR (95% CI)	*P*	OR (95% CI)	*P*	OR (95% CI)	*P*
*CARD8*	Control	126 (60.6)	71 (34.1)	11 (5.3)	1.00				1.00			
(rs1965759)	APN	70 (53.8)	59 (45.4)	1 (0.8)	1.32 (0.85, 2.05)	0.223	1.00		**7.20 (0.92, 56.5)**	**0.033**	1.00	
	ALN	116 (54.7)	87 (41.0)	9 (4.2)	1.27 (0.86, 1.87)	0.224	0.97 (0.62, 1.50)	0.875	1.26 (0.51, 3.11)	0.616	1.00 (0.02, 1.42)	0.096
	Combined	186 (54.4)	146 (33.9)	10 (2.9)	1.29 (0.91, 1.83)	0.155			1.85 (0.77, 4.44)	0.161		
		TT	TA	AA	OR (95% CI)	*P*	OR (95% CI)	*P*	OR (95% CI)	*P*	OR (95% CI)	*P*
*CARD8*	Control	65 (29.4)	108 (48.9)	48 (21.7)	1.00				1.00			
(rs2043211)	APN	37 (28.0)	72 (54.5)	23 (17.4)	1.07 (0.66, 1.73)	0.782	1.00		1.32 (0.76, 2.28)	0.330	1.00	
	ALN	58 (26.5)	118 (53.9)	43 (19.6)	1.16 (0.77, 1.77)	0.475	1.09 (0.67, 1.77)	0.733	1.14 (0.72, 1.81)	0.573	0.87 (0.50, 1.52)	0.622
	Combined	95 (27.1)	190 (54.1)	66 (18.8)	1.13 (0.78, 1.64)	0.529			1.20 (0.79, 1.82)	0.386		
		GG	GA	AA	OR (95% CI)	*P*	OR (95% CI)	*P*	OR (95% CI)	*P*	OR (95% CI)	*P*
*CARD8*	Control	113 (51.6)	93 (42.5)	13 (5.9)	1.00				1.00			
(rs1972619)	APN	60 (48.0)	53 (42.4)	12 (9.6)	1.16 (0.74, 1.79)	0.521	1.00		0.59 (0.26, 1.35)	0.208	1.00	
	ALN	93 (44.5)	97 (46.4)	19 (9.1)	1.33 (0.91, 1.95)	0.142	1.15 (0.74, 1.80)	0.534	0.63 (0.30, 1.31)	0.215	1.06 (0.50, 2.27)	0.877
	Combined	153 (45.8)	150 (44.9)	31 (9.3)	1.26 (0.90, 1.77)	0.183			0.62 (0.32, 1.21)	0.155		
		CC	CT	TT	OR (95% CI)	*P*	OR (95% CI)	*P*	OR (95% CI)	*P*	OR (95% CI)	*P*
*IL1-β*	Control	52 (23.7)	122 (55.7)	45 (20.5)	1.00				1.00			
(rs1143629)	APN	24 (18.0)	71 (53.4)	38 (28.6)	1.43 (0.83, 2.45)	0.196	1.00		0.65 (0.40, 1.08)	0.093	1.00	
	ALN	52 (24.0)	114 (52.5)	51 (23.5)	1.00 (0.64, 1.55)	1.000	0.70 (0.41, 1.20)	0.196	0.85 (0.54, 1.34)	0.488	1.30 (0.80, 2.13)	0.287
	Combined	76 (21.7)	185 (52.4)	89 (25.4)	1.14 (0.76, 1.70)	0.537			0.77 (0.51, 1.15)	0.200		

^a^ *P*-values <0.05 are shown in bold.

^b^ APN + ALN

^c^ Statistically significant with correction for multiple-SNP testing (*P* < 0.0055)

**Table 2:** Allele frequency analysis of the SNPs.

SNP	Major allele frequency (%)				APN *vs*. control		ALN *vs*. control		Combined *vs*. control		ALN *vs*. APN	
	Control	APN	ALN	Combined^b^	OR (95% CI)	*P* ^a^	OR (95% CI)	*P* ^a^	OR (95% CI)	*P* ^a^	OR (95% CI)	*P* ^a^
*NLRP3*, (rs4612666), C major allele	55.53	48.87	57.49	54.12	0.77 (0.56, 1.04)	0.089	1.08 (0.82, 1.43)	0.569	0.95 (0.74, 1.21)	0.649	**1.42 (1.04, 1.93)**	**0.028**
*NLRP3*, (rs4925650), G major allele	55.31	57.25	51.17	53.48	1.08 (0.79, 1.48)	0.621	0.85 (0.65, 1.11)	0.228	0.93 (0.73, 1.19)	0.553	0.78 (0.57, 1.07)	0.120
*NLRP3*, (rs10754558), C major allele	61.38	69.78	63.72	66.05	**1.46 (1.02, 2.01)**	**0.021**	1.11 (0.84, 1.45)	0.475	1.23 (0.96, 1.57)	0.104	0.76 (0.55, 1.05)	0.095
*NLRP3*, (rs1539019), G major allele	63.30	62.60	57.21	59.29	0.97 (0.71, 1.33)	0.851	0.78 (0.59, 1.02)	0.069	0.84 (0.66, 1.08)	0.181	0.80 (0.58, 1.10)	0.165
*NLRP3*, (rs4925663), T major allele	53.55	50.43	49.27	49.69	0.88 (0.64, 1.22)	0.448	0.84 (0.64, 1.11)	0.224	0.86 (0.67, 1.10)	0.227	0.96 (0.69, 1.32)	0.778
*CARD8*, (rs1965759), C major allele	77.64	76.54	75.24	75.73	0.94 (0.65, 1.36)	0.739	0.88 (0.64, 1.20)	0.411	0.90 (0.69, 1.20)	0.468	0.93 (0.65, 1.34)	0.700
*CARD8*, (rs2043211), T major allele	53.84	55.30	53.42	54.13	1.06 (0.78, 1.44)	0.707	0.98 (0.75, 1.28)	0.896	1.01 (0.80, 1.29)	0.928	0.93 (0.68, 1.26)	0.625
*CARD8*, (rs1972619), G major allele	72.83	69.20	67.70	68.26	0.84 (0.60, 1.18)	0.310	0.78 (0.58, 1.05)	0.101	0.80 (0.62, 1.05)	0.105	0.93 (0.67, 1.31)	0.688
*IL1-β*, (rs1143629), C major allele	51.60	44.74	50.23	48.14	0.76 (0.56, 1.03)	0.078	0.95 (0.73, 1.23)	0.685	0.87 (0.69, 1.11)	0.258	1.25 (0.92, 1.69)	0.159

^a^ *P*-values <0.05 are shown in bold.

^b^ APN + ALN

As VUR has been indicated as a significant risk factor for upper UTIs [9, 23], further analyses were carried out in the subgroups of APN and ALN patients with no VUR. VUR was not observed in 65 APN patients (35 males and 30 females; 2.51 ± 2.77 years old) and 133 ALN patients (67 males and 66 females; 2.92 ± 2.63 years old).Our previous report indicated that the number of patients with VUR in the control group needed to be determined based upon the reported prevalence rate because voiding cystourethrography (VCUG) was not medically advised in these control patients [15, 44]. Because the prevalence rate of VUR at the mean age of the control group (2.96 years old) was ~0.3%, the number of patients with VUR in the 225 control cases could be assumed to be zero. Additionally, no differences were found in age and sex ratio among the control and non-VUR subgroup of APN and ALN.

Among the nine SNPs examined using the dominant model, only *NLRP3* (rs4612666) showed a significant difference in genotypic frequency between the control group and non-VUR in the APN, ALN, and combined (APN+ALN) subgroups. In these analyses, the CC genotype frequency of the non-VUR subgroups of APN, ALN, and APN+ALN cases were significantly lower than that of the control (Table 3). However, with the correction for multiple-SNP testing, only the non-VUR subgroup of the APN+ALN group remained significantly different from the control group (*P* < 0.0055). Nevertheless, allele frequency analyses showed no significant differences between the control and non-VUR subgroups of APN and ALN (Table 4).

**Table 3:** Genotypic analysis of the SNPs in the patient subgroup without vesicoureteral reflux (non-VUR).

SNP	Group	Genotype, *n* (%)			Dominant model				Recessive model			
		00	01	11	(01, 11 *vs*. 00)				(00, 01*vs*. 11)			
		CC	CT	TT	OR (95% CI)	*P* ^a^	OR (95% CI)	*P* ^a^	OR (95% CI)	*P* ^a^	OR (95% CI)	*P* ^a^
*NLRP3*	Control	68 (32.7)	95 (45.7)	45 (21.6)	1.00				1.00			
(rs4612666)	APN	12 (19.0)	40 (63.5)	11 (17.5)	**2.06 (1.03, 4.13)**	**0.038**	1.00		1.31 (0.63, 2.71)	0.473	1.00	
	ALN	26 (20.6)	82 (65.1)	18 (14.3)	**1.87 (1.11, 3.14)**	**0.018**	0.91 (0.42, 1.94)	0.797	1.66 (0.91, 3.01)	0.096	1.27 (0.56, 2.88)	0.568
	Combined^b^	38 (20.1)	122 (64.6)	29 (15.3)	**1.93 (1.22, 3.05)**	**0.005** ^c^			1.52 (0.91, 2.55)	0.108		
		GG	GA	AA	OR (95% CI)	*P*	OR (95% CI)	*P*	OR (95% CI)	*P*	OR (95% CI)	*P*
*NLRP3*	Control	66 (31.9)	97 (46.9)	44 (21.3)	1.00				1.00			
(rs4925650)	APN	20 (32.8)	34 (55.7)	7 (11.5)	0.96 (0.52, 1.77)	0.894	1.00		2.08 (0.89, 4.90)	0.087	1.00	
	ALN	43 (33.1)	59 (45.4)	28 (21.5)	0.95 (0.59, 1.51)	0.820	0.99 (0.52, 1.89)	0.968	0.98 (0.58, 1.68)	0.951	0.47 (0.19, 1.15)	0.094
	Combined	63 (33.0)	93 (48.7)	35 (18.3)	0.95 (0.63, 1.45)	0.815			1.20 (0.73, 1.97)	0.464		
		CC	CG	GG	OR (95% CI)	*P*	OR (95% CI)	*P*	OR (95% CI)	*P*	OR (95% CI)	*P*
*NLRP3*	Control	85 (37.9)	105 (46.9)	34 (15.2)	1.00				1.00			
(rs10754558)	APN	28 (45.9)	25 (41.0)	8 (13.1)	0.72 (0.41, 1.28)	0.260	1.00		1.19 (0.52, 2.71)	0.687	1.00	
	ALN	45 (34.9)	63 (48.8)	21 (16.3)	1.14 (0.73, 1.79)	0.566	1.58 (0.85, 2.95)	0.145	0.92 (0.51, 1.67)	0.784	0.78 (0.32, 1.87)	0.571
	Combined	73 (38.4)	88 (46.3)	29 (15.3)	0.98 (0.66, 1.46)	0.921			0.99 (0.58, 1.70)	0.981		
		GG	GT	TT	OR (95% CI)	*P*	OR (95% CI)	*P*	OR (95% CI)	*P*	OR (95% CI)	*P*
*NLRP3*	Control	86 (39.4)	104 (47.7)	28 (12.8)	1.00				1.00			
(rs1539019)	APN	20 (31.7)	33 (52.4)	10 (15.9)	1.40 (0.77, 2.54)	0.267	1.00		0.78 (0.36, 1.71)	0.682	1.00	
	ALN	46 (35.9)	63 (49.2)	19 (14.8)	1.16 (0.74, 1.83)	0.516	0.83 (0.44, 1.58)	0.567	0.85 (0.45, 1.59)	0.600	1.08 (0.47, 2.49)	0.852
	Combined	66 (34.6)	96 (50.3)	29 (15.2)	1.23 (0.82, 1.85)	0.307			0.82 (0.47, 1.44)	0.496		
		TT	TC	CC	OR (95% CI)	*P*	OR (95% CI)	*P*	OR (95% CI)	*P*	OR (95% CI)	*P*
*NLRP3*	Control	57 (28.9)	97 (49.2)	43 (21.8)	1.00				1.00			
(rs4925663)	APN	15 (26.8)	27 (48.2)	14 (25.0)	1.11 (0.57, 2.17)	0.753	1.00		0.84 (0.42, 1.68)	0.616	1.00	
	ALN	35 (27.8)	57 (45.2)	34 (27.0)	1.06 (0.64, 1.74)	0.822	0.95 (0.47, 1.93)	0.890	0.76 (0.45, 1.27)	0.289	0.90 (0.44, 1.86)	0.779
	Combined	50 (27.5)	84 (46.2)	48 (26.4)	1.08 (0.69, 1.68)	0.752			0.78 (0.49, 1.25)	0.301		
		CC	CT	TT	OR (95% CI)	*P*	OR (95% CI)	*P*	OR (95% CI)	*P*	OR (95% CI)	*P*
*CARD8*	Control	126 (60.6)	71 (34.1)	11 (5.3)	1.00				1.00			
(rs1965759)	APN	37 (60.7)	23 (37.7)	1 (1.6)	1.00 (0.56, 1.79)	0.991	1.00		3.35 (0.42, 26.5)	0.309	1.00	
	ALN	72 (55.8)	50 (38.8)	7 (5.4)	1.22 (0.78, 1.90)	0.388	1.22 (0.66, 2.27)	0.529	0.97 (0.37, 2.58)	0.956	0.29 (0.04, 2.44)	0.440
	Combined	109 (57.4)	73 (38.4)	8 (4.2)	1.14 (0.77, 1.70)	0.516			1.27 (0.50, 3.23)	0.788		
		TT	TA	AA	OR (95% CI)	*P*	OR (95% CI)	*P*	OR (95% CI)	*P*	OR (95% CI)	*P*
*CARD8*	Control	65 (29.4)	108 (48.9)	48 (21.7)	1.00				1.00			
(rs2043211)	APN	17 (26.6)	34 (53.1)	13 (20.3)	1.15 (0.62, 2.15)	0.657	1.00		1.09 (0.55, 2.17)	0.809	1.00	
	ALN	38 (29.2)	65 (50.0)	27 (20.8)	1.01 (0.63, 1.62)	0.971	0.88 (0.45, 1.71)	0.698	1.06 (0.62, 1.80)	0.834	0.97 (0.46, 2.04)	0.941
	Combined	55 (28.4)	99 (51.0)	40 (20.6)	1.05 (0.69, 1.61)	0.812			1.07 (0.67, 1.71)	0.784		
		GG	GA	AA	OR (95% CI)	*P*	OR (95% CI)	*P*	OR (95% CI)	*P*	OR (95% CI)	*P*
*CARD8*	Control	113 (51.6)	93 (42.5)	13 (5.9)	1.00				1.00			
(rs1972619)	APN	28 (50.9)	23 (41.8)	4 (7.3)	1.03 (0.57, 1.86)	0.927	1.00		0.81 (0.25, 2.57)	0.713	1.00	
	ALN	59 (46.8)	56 (44.4)	11 (8.7)	1.21 (0.78, 1.88)	0.393	1.18 (0.63, 2.22)	0.613	0.66 (0.29, 1.52)	0.326	0.82 (0.25, 2.70)	0.744
	Combined	87 (48.1)	79 (43.6)	15 (8.3)	1.15 (0.78, 1.71)	0.482			0.70 (0.32, 1.51)	0.359		
		CC	CT	TT	OR (95% CI)	*P*	OR (95% CI)	*P*	OR (95% CI)	*P*	OR (95% CI)	*P*
*IL1-β*	Control	52 (23.7)	122 (55.7)	45 (20.5)	1.00				1.00			
(rs1143629)	APN	12 (20.0)	34 (56.7)	14 (23.3)	1.25 (0.62, 2.52)	0.541	1.00		0.85 (0.43, 1.68)	0.640	1.00	
	ALN	33 (25.8)	67 (52.3)	28 (21.9)	0.90 (0.54, 1.48)	0.670	0.72 (0.34, 1.52)	0.387	0.92 (0.54, 1.57)	0.770	1.09 (0.52, 2.26)	0.823
	Combined	45 (23.9)	101 (53.7)	42 (22.3)	0.99 (0.63, 1.56)	0.964			0.90 (0.56, 1.45)	0.660		

^a^ *P-*values <0.05 are shown in bold.

^b^ APN + ALN

^c^ Statistically significant with correction for multiple-SNP testing (*P* < 0.0055)

**Table 4:** Allele frequency analysis of the SNPs in the non-VUR patient subgroup.

SNP	Major allele frequency (%)				APN *vs*. control		ALN *vs*. control		Combined *vs*. control		ALN *vs*. APN	
	Control	APN	ALN	Combined^b^	OR (95% CI)	*P* ^a^	OR (95% CI)	*P* ^a^	OR (95% CI)	*P* ^a^	OR (95% CI)	*P* ^a^
*NLRP3*, (rs4612666), C major allele	55.53	50.79	53.17	52.38	0.83 (0.56, 1.23)	0.350	0.91 (0.66, 1.25)	0.554	0.88 (0.67, 1.17)	0.374	1.10 (0.72, 1.69)	0.662
*NLRP3*, (rs4925650), G major allele	55.31	60.66	55.77	57.33	1.25 (0.83, 1.88)	0.296	1.02 (0.75, 1.39)	0.908	1.09 (0.82, 1.44)	0.567	0.82 (0.53, 1.27)	0.368
*NLRP3*, (rs10754558), C major allele	61.38	66.39	59.30	61.58	1.24 (0.82, 1.89)	0.311	0.92 (0.67, 1.25)	0.586	1.10 (0.76, 1.34)	0.954	0.74 (0.47, 1.16)	0.185
*NLRP3*, (rs1539019), G major allele	63.30	57.94	60.55	59.69	0.80 (0.53, 1.20)	0.274	0.89 (0.65, 1.22)	0.470	0.86 (0.65, 1.14)	0.289	1.11 (0.72, 1.72)	0.625
*NLRP3*, (rs4925663), T major allele	53.55	50.89	50.40	50.55	0.90 (0.59, 1.37)	0.619	0.88 (0.64, 1.21)	0.433	0.89 (0.67, 1.18)	0.408	0.98 (0.63, 1.53)	0.930
*CARD8*, (rs1965759), C major allele	77.64	79.51	75.19	76.58	1.12 (0.68, 1.84)	0.662	0.87 (0.61, 1.26)	0.464	0.94 (0.68, 1.31)	0.721	0.78(0.46, 1.32)	0.354
*CARD8*, (rs2043211), T major allele	53.84	53.13	54.23	53.87	0.97 (0.66, 1.44)	0.885	1.02 (0.75, 1.38)	0.921	1.00 (0.76, 1.32)	0.995	1.05 (0.68, 1.60)	0.837
*CARD8*, (rs1972619), G major allele	72.83	71.82	69.05	69.89	0.95 (0.60, 1.51)	0.831	0.83 (0.59, 1.17)	0.89	0.87 (0.64, 1.18)	0.359	0.88 (0.53, 1.43)	0.597
*IL1-β*, (rs1143629), C major allele	51.60	48.33	51.95	50.80	0.88 (0.59, 1.32)	0.526	1.01 (0.75, 1.38)	0.928	0.97 (0.74, 1.28)	0.820	1.16 (0.75, 1.78)	0.513

^a^ *P-*values <0.05 are shown in bold.

^b^ APN + ALN

## Discussion

A concerted host innate and adaptive immune response is required for defending against bacterial invasion or infection. For this defense, the host must first recognize the pathogen through the expression of various extracellular or intracellular PRRs such as the TLRs and the NOD-like receptors (NLRs). Following this microbial sensing step, consecutive activation of intracellular signaling cascades marks the initiation of the immune responses [13]. These responses determine the host health status as well as the clinical severity [9]. Therefore, the risk of bacterial infection would be influenced by the signal transmission effectiveness in the immune response pathway. Defective signal transmission caused by a genetic polymorphism in the receptors, adaptors, or cytokines would influence an individual’s risk for infectious diseases [11, 14, 15, 45, 46].

NLRs have been considered important PRRs for various PAMPs, including lipopolysaccharide (LPS), bacterial toxins, and viral nucleic acids [19, 22]. The NLRs can also sense damage-associated molecular patterns (DAMPs) such as potassium efflux, reactive oxygen species, urate crystals, and extracellular matrix components including hyaluronan and biglycan [13, 16–20]. Following recognition of the signal pattern, the inflammasome consisting of NLRs, adaptor protein ASC, and caspase–1 is activated, and cytokines are released. These signal transmission cascades form an inflammasome-dependent innate immunity pathway.

In the present study, the most significant finding was the association between *NLRP3* (rs4612666) and severe renal parenchymal infections, APN and ALN. After excluding patients with VUR, a well-known risk factor for severe UTI, we found that patients with APN and ALN had a lower genotype frequency of *NLRP3* (rs4612666) CC than did controls. This result implies that the CC genotype in *NLRP3* (rs4612666) participates in regulating the inflammatory responses for pediatric renal parenchymal infections in patients from Taiwan.

Genetic variations can modify the extent or severity of disease in susceptible patients. Mutations in the NLRP3 gene have been linked to several autoinflammatory disorders including Muckle–Wells syndrome, familial cold autoinflammatory syndrome, and chronic infantile neurologic cutaneous and articular syndrome [17]. Additionally, *NLRP3* polymorphisms have been associated with susceptibility to a number of different diseases, such as coal workers' pneumoconiosis, Crohn’s disease, sporadic malignant melanoma, psoriasis and many others [27, 28, 30–32, 34, 37, 41, 42, 47]. Inflammasome activation has been associated with microbial sensing and reaction to various microbial infections [13, 16, 21, 22]. However, to our knowledge, no study has reported the likely association of renal parenchymal infections and the inflammasome-dependent innate immune pathway. This investigation suggested that the *NLRP3* polymorphisms could influence the susceptibility of pediatric patients to severe renal parenchymal infections.

NLRP3 is in the NLR family of proteins. Other NLR proteins, including NLRP1, NLRC4 and AIM2 are also involved in sensing and activation by various microbes [16, 17, 21, 22]. Whether polymorphisms in genes encoding other NLR proteins are linked with the severity of pediatric renal parenchymal infections remains to be explored. Similarly, the likely roles of IL–18 polymorphism, another cytokine indicated in inflammasome-dependent innate immunity pathway in various kidney diseases [17–20], in pediatric renal infectious diseases would also require further investigations.

Previous study has shown the CC genotype frequency is higher in the cases with food-induced anaphylaxis than those without [42]. In addition, the NLRP3 rs4162666 C allele has been demonstrated to be associated with higher transcriptional activity in THP–1 cell *in vitro* [42]. However, the CC genotype is significantly less in the non-VUR subgroup of cases with APN and ALN as compared to the control. This suggests that the finding about the higher transcription activity in C allele may not be feasible to explain our results here. More experimental works are warranted to clarify about the possible biological pathway.

In summary, this is the first study to show that the inflammasome-dependent innate immunity pathway is likely involved in severe renal parenchymal infection. We showed that genetic polymorphisms in *NLRP3* (rs4612666) are related to the pediatric patient’s susceptibility to APN and ALN. Further investigations of phenotypes or functional assessment of this SNP is warranted to confirm their pathogenic roles. Additionally, as the statistical power to detect significant associations with genetic variants is determined by sample size, a larger cohort study is recommended to replicate and validate the associations of SNPs with the severe UTIs, including APN and ALN.

## Supporting Information

S1 File The supplementary patient data. (XLSX)

S1 Table Primers employed for DNA amplification by direct DNA sequencing analysis of single nucleotide polymorphisms (SNPs). (DOCX)

S2 Table Primers used for DNA amplification and mini-sequencing analysis of the SNPs. (DOCX)
