# Supplementary material for: Genetic Polymorphisms in Inflammasome-Dependent Innate Immunity among Pediatric Patients with Severe Renal Parenchymal Infections
Source: PLoS One. 2015 Oct 7;10(10):e0140128. doi: 10.1371/journal.pone.0140128 (PMC4596571; doi:10.1371/journal.pone.0140128)
Supplement: S1 Table — (DOCX) [file pone.0140128.s002.docx]

S1 Table. Primers employed for DNA amplification by direct DNA sequencing analysis of single nucleotide polymorphisms (SNPs)

| Position | Primer sequence (5′→3′, forward) | Primer sequence (5′→3′, reverse) | Annealing temperature |
| --- | --- | --- | --- |
| *NLRP3* (rs4612666), C>T | CCAAACGTCCTTCAACGG | GGGAGTGGACACAAGCTC | 55 °C |
| *NLRP3* (rs4925650), A>G | CAGCACTTTGTGAGGCTGA | CCAAACTCCACGGTTCTTC | 55 °C |
| *NLRP3* (rs10754558), C>G | TCTTGGTAGGAGTGGAAACG | AGCGGGAATGATGATATGAG | 55 °C |
| *CARD8* (rs1965759), C>T | CTGCAGAATGGCAAAAGAGC | CTTTACCCGCTGAGCATTGT | 55 °C |
| *CARD8* (rs2043211), A>T | AACCCACTGGTCTTCGATTG | GGTGAGATGGAGTCGTAGGG | 56 °C |
| *IL1-β* (rs1143629), C>T | GAGCTCGCCAGTGAAATGAT | AAACAGCCTGCCTCTCAAAG | 55 °C |
