# Supplementary material for: Genetic Polymorphisms in Inflammasome-Dependent Innate Immunity among Pediatric Patients with Severe Renal Parenchymal Infections
Source: PLoS One. 2015 Oct 7;10(10):e0140128. doi: 10.1371/journal.pone.0140128 (PMC4596571; doi:10.1371/journal.pone.0140128)
Supplement: S2 Table — (DOCX) [file pone.0140128.s003.docx]

S2 Table. Primers used for DNA amplification and mini-sequencing analysis of the SNPs

| Position | Primer sequence (5′→3′) | PCR product size | Mini-sequencing primer sequence (5′→3′) | Molecular weight of mini-sequencing product |
| --- | --- | --- | --- | --- |
| *NLRP3* (rs1539019), G>T | Sense: CTT GGG AGG TCA CAG ACG TT | 155 bp | GAG TGT CCT TGG ACA AAG | Primer: 5563.63 |
|  | Anti-sense: CAG TTG GCT TCA GAA AGA TGC |  |  | G allele: 6181.03 |
|  |  |  |  | T allele: 5851.83 |
| *NLRP3* (rs4925663), C>T | Sense: AGG GAG ACG ATC ATC AGG TG | 179 bp | CTG CCC GGG CAA TAA AG | Primer: 5204.4 |
|  | Anti-sense: GAC ACC GCT GTG AAT GAC AC |  |  | C allele: 5477.59 |
|  |  |  |  | T allele: 5781.78 |
| *CARD8* (rs1972619), A>G | Sense: CAA ATG GAA GCA TTG TGT GTG | 183 bp | GGA GGT TTT TAA ACC ATT T | Primer: 5832.81 |
|  | Anti-sense: ACT GTG TTT GGG GAG CAG AC |  |  | A allele: 6434.21 |
|  |  |  |  | G allele: 6146.02 |
